# Region-Functionalized Laser-Induced Graphene Sensor Array for Flexible and Intelligent Multi-Gas Detection

**DOI:** 10.3390/nano16201321

**Published:** 2026-10-20

**Authors:** Wengan Wang, Chaoqi Zhang, Guiyong Chen, Peihao Xu, Fan Wu, Misheng Liang, Rui You

**Affiliations:** 1 AVIC Chengdu Aircraft Industrial (Group) Co., Ltd., Chengdu 610092, China; 2 School of Instrument Science and Opto-Electronics Engineering, Beijing Information Science and Technology University, Beijing 100192, China; zcq18330172237@163.com (C.Z.); fanwoo@foxmail.com (F.W.); 3 Laboratory of Intelligent Microsystems, Beijing Information Science and Technology University, Beijing 100192, China

**Keywords:** laser-induced graphene, flexible gas sensor, gas sensor array, functional nanomaterials, multi-gas detection, machine learning

## Abstract

Flexible multi-gas sensor array has promising applications in wearable monitoring, environmental safety and intelligent sensing systems. In this work, a flexible 2 × 2 multi-gas sensor array based on region-functionalized laser-induced graphene (LIG) was developed for room-temperature gas detection and intelligent identification. MXene, MoS_2_, TiO_2_ and CuO were selectively introduced into different regions of a polyimide substrate, followed by femtosecond pulsed-laser direct writing to form four functionally differentiated LIG-based sensing units. The porous LIG network simultaneously serves as the conductive pathway, gas-adsorption interface, and sensing electrode, enabling the integration of multiple sensing functions within a single flexible platform. The gas-sensing results show that the array exhibits differential response characteristics toward NH_3_, H_2_S, acetone, and ethanol. LIG@MXene exhibited a pronounced and repeatable response toward NH_3_, whereas LIG@MoS_2_, LIG@TiO_2_, and LIG@CuO showed preferential responses toward H_2_S, acetone, and ethanol, respectively. The distinct response amplitudes and polarities of the four sensing channels provide multidimensional features for gas-condition identification. The device also maintained relatively stable NH_3_ sensing performance under mechanical bending, with a response retention of approximately 84.2% at a bending angle of 120°. Based on the multidimensional response signals of the four-channel array, a backpropagation neural network (BPNN) was employed for gas-condition identification. The overall correlation coefficient between the network outputs and the numerically encoded target labels reached 0.99974 for the single-gas dataset and 0.94894 for the mixed-gas dataset. This study provides an effective strategy for integrating region-specific material functionalization, flexible laser fabrication, multi-channel sensing, and intelligent gas identification within a single LIG-based sensing platform.

## 1. Introduction

With the rapid development of the Internet of Things, intelligent healthcare, environmental monitoring, and industrial safety, gas sensors play an increasingly important role in environmental pollution monitoring, human breath analysis, food safety detection, and toxic and harmful gas warning. Although traditional rigid gas sensors can achieve effective gas detection, their poor mechanical flexibility makes them difficult to adapt to complex application scenarios involving human skin, textiles, and curved surfaces [1,2]. At the same time, some rigid array devices involve complex fabrication processes and considerable integration difficulties, which limit their further application in wearable monitoring and intelligent sensing systems. In contrast, flexible gas sensors offer advantages such as lightweight construction, mechanical flexibility, conformability, and ease of integration, and can play an important role in the detection of human respiratory gases, environmental pollutants, and volatile organic compounds [3]. In particular, functional nanomaterials, such as graphene, MXene, transition metal sulfides, and metal oxides, have attracted extensive attention for constructing high-performance flexible gas sensors because of their high specific surface areas, favorable electrical properties, and compatibility with flexible devices [4].

Graphene and its derived materials have shown considerable potential for the detection of NH_3_, NO_2_, and volatile organic compounds due to their high carrier mobility, rich surface active sites, and room-temperature gas-sensing capability [5,6]. The gas-sensing response of graphene mainly originates from gas adsorption-induced charge transfer and the resulting modulation of carrier concentration. Through defect engineering, surface functionalization, and decoration with metal or metal-oxide nanophases, the sensitivity, selectivity, and response–recovery characteristics of graphene-based gas sensors can be effectively improved [7,8]. However, the effect of surface functionalization or doping is strongly dependent on both the functional material and the target gas species, and the experimentally observed response may not always follow predictions based solely on the intrinsic properties or work function of the dopant. For example, Kwak et al. reported that metal nanoparticle functionalization of flexible LIG enhanced its response toward the reducing gas NH_3_ but produced a different response trend toward the oxidizing gas NO_2_, highlighting the combined roles of additional active sites, surface chemical states, and interfacial charge transfer in determining the sensing behavior [9]. In recent years, graphene-based gas sensors have gradually developed from simple coating configurations toward porous structures, textile-integrated devices, printed devices, and LIG-based structures. Their application scenarios have also been extended to personal exposure monitoring, breath analysis, industrial safety warning, and smart textiles [10].

LIG provides an attractive fabrication route for flexible gas sensors because porous graphene-like carbon structures can be formed directly on carbon-containing precursors such as polyimide (PI) through laser irradiation. This mask-free direct-writing process offers rapid fabrication, high patterning flexibility, and good compatibility with flexible substrates [11]. A femtosecond pulsed laser was employed for the direct writing of LIG on functionalized PI films. The formation of LIG is mainly associated with localized photothermal conversion induced by the highly concentrated energy deposition of the ultrashort laser pulses. The resulting rapid heating promotes bond cleavage and rearrangement, followed by carbonization and graphitization of the PI precursor. The short laser–material interaction time also helps suppress excessive heat diffusion into the surrounding region and facilitates precise pattern formation. The morphology, defect density, and graphitization degree of the resulting LIG can be regulated by processing parameters such as laser power and scanning speed. Compared with conventional graphene-device fabrication involving material transfer, lithography, and additional post-processing, laser direct writing can simultaneously construct conductive pathways, porous sensing interfaces, and patterned electrodes on flexible substrates, thereby simplifying device fabrication [12]. Moreover, the three-dimensional porous conductive skeleton of LIG provides abundant gas adsorption sites and facilitates molecular diffusion, while its microstructure can be further tailored through laser-processing parameters, providing a suitable platform for flexible room-temperature gas sensing [13].

Recent developments in flexible gas sensing have increasingly shifted from single sensing elements toward multi-sensor arrays combined with pattern-recognition and machine-learning algorithms. Flexible multi-gas sensing arrays can generate differentiated response patterns by integrating sensing units with distinct gas-response characteristics on a common flexible substrate. These multidimensional signals can subsequently be analyzed using principal component analysis, linear discriminant analysis, support vector machines, or neural networks to improve gas discrimination [14,15]. In parallel, considerable progress has been made in LIG-based flexible gas sensors. Yang et al. developed a moisture-resistant and stretchable LIG-based NOx gas sensor for environmental monitoring and breath analysis, achieving ppb-level detection together with stable sensing performance under mechanical deformation [16]. Lim et al. prepared UV-LIG devices on PI films using a pulsed ultraviolet laser and further integrated MOFs as functional sensing materials, achieving ppb-level room-temperature gas detection while retaining stable performance under mechanical bending [17]. Beyond individual gas detection, the combination of differentiated sensing materials, sensor arrays, and intelligent algorithms provides an effective route for addressing the limited selectivity of individual sensing elements. For example, Moon et al. constructed a 2 × 2 flexible gas sensing array based on rGO and different metal oxide nanoparticles and successfully distinguished NO_2_, NO, and SO_2_ using a neural-network-based recognition model, demonstrating the potential of combining flexible sensor arrays with machine learning for complex gas identification [18]. More recently, Cho et al. developed an AI-assisted artificial olfactory system by integrating multidimensional gas-sensing characteristics with deep-learning algorithms, further demonstrating the potential of combining sensor arrays with intelligent data analysis for reliable multi-gas identification [19].

Despite these advances, the integration of multiple functionally differentiated sensing materials within a flexible LIG platform for multi-gas identification remains insufficiently explored. In particular, achieving region-specific functionalization while maintaining a simple fabrication process and generating distinguishable multi-channel response patterns remains challenging. In this work, we develop a flexible 2 × 2 multi-gas sensor array for the room-temperature detection and identification of NH_3_, H_2_S, acetone, and ethanol. MXene, MoS_2_, TiO_2_, and CuO are selectively introduced into different regions of a PI substrate, followed by femtosecond pulsed-laser direct writing to form four functionally differentiated LIG-based sensing units. The resulting array produces distinct multi-channel response patterns, which are further analyzed using a BPNN for gas identification. The main novelty of this work lies in combining region-specific material functionalization, in situ LIG formation, flexible multi-channel sensing, and intelligent gas identification within a single integrated sensing platform.

## 2. Materials and Methods

### 2.1. Sensor Fabrication

**Scheme 1:**
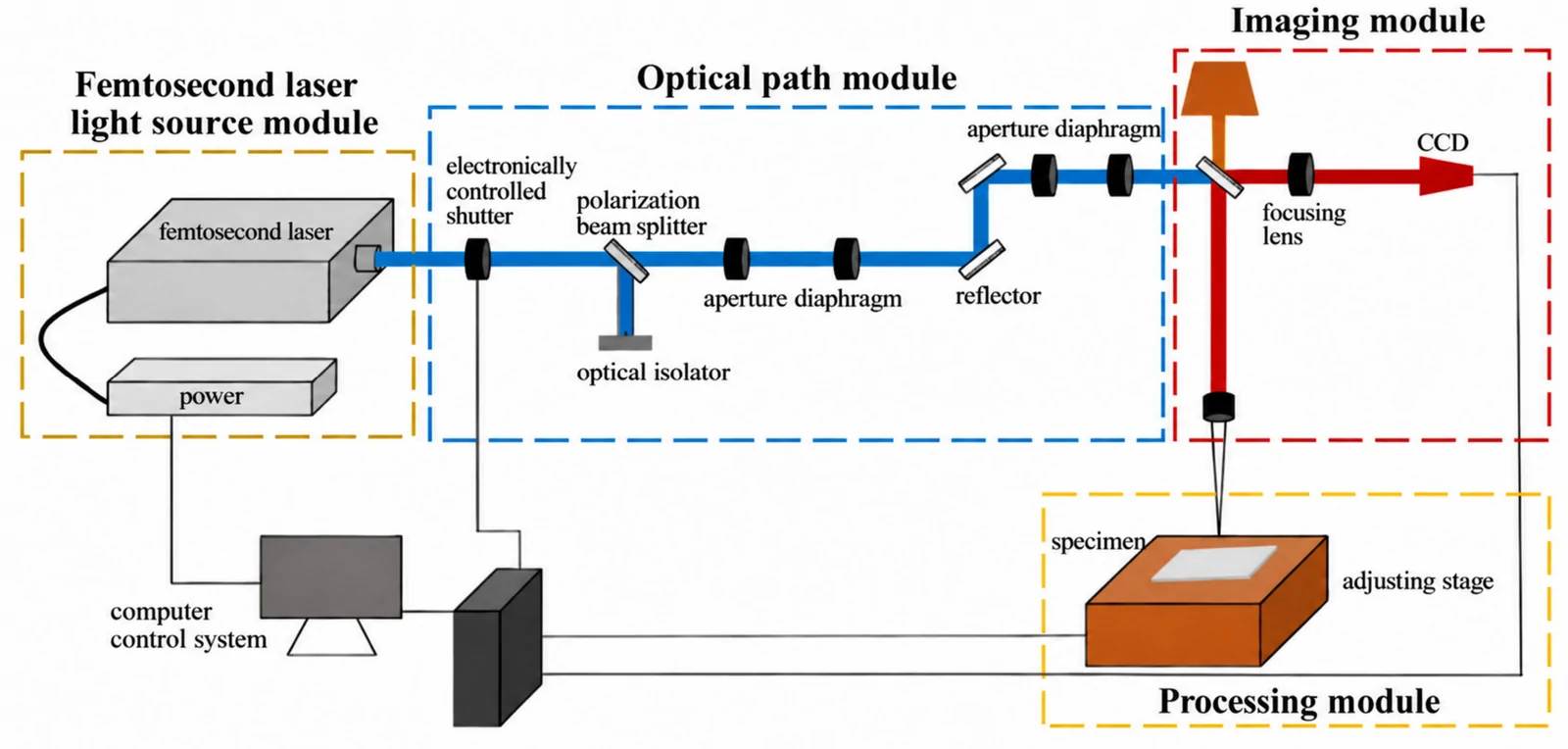
Schematic illustration of the femtosecond laser processing system used for sensor fabrication.

The femtosecond laser processing system used in this study is schematically illustrated in Scheme 1. The femtosecond laser (Hangzhou Yacto Technology Co., Ltd., Hangzhou, China) operated at a wavelength of 1030 nm with a pulse duration of 137 fs and a repetition frequency of 200 kHz, and was focused onto the sample surface using a 10× Mitutoyo M Plan Apo NIR objective lens with a numerical aperture (NA) of 0.3. The system consists of a femtosecond laser source module, an optical path module, an imaging module, a processing module, and a computer control system. The optical path module contains an electronically controlled shutter, a polarization beam splitter, an optical isolator, aperture diaphragms, and reflectors for controlling and guiding the laser beam. The imaging module, consisting mainly of a focusing lens and a CCD camera, enables observation of the processing region, while the processing module provides specimen positioning and motion control during laser direct writing. The computer control system coordinates the laser output and the motion of the processing stage.

**Figure 1:**
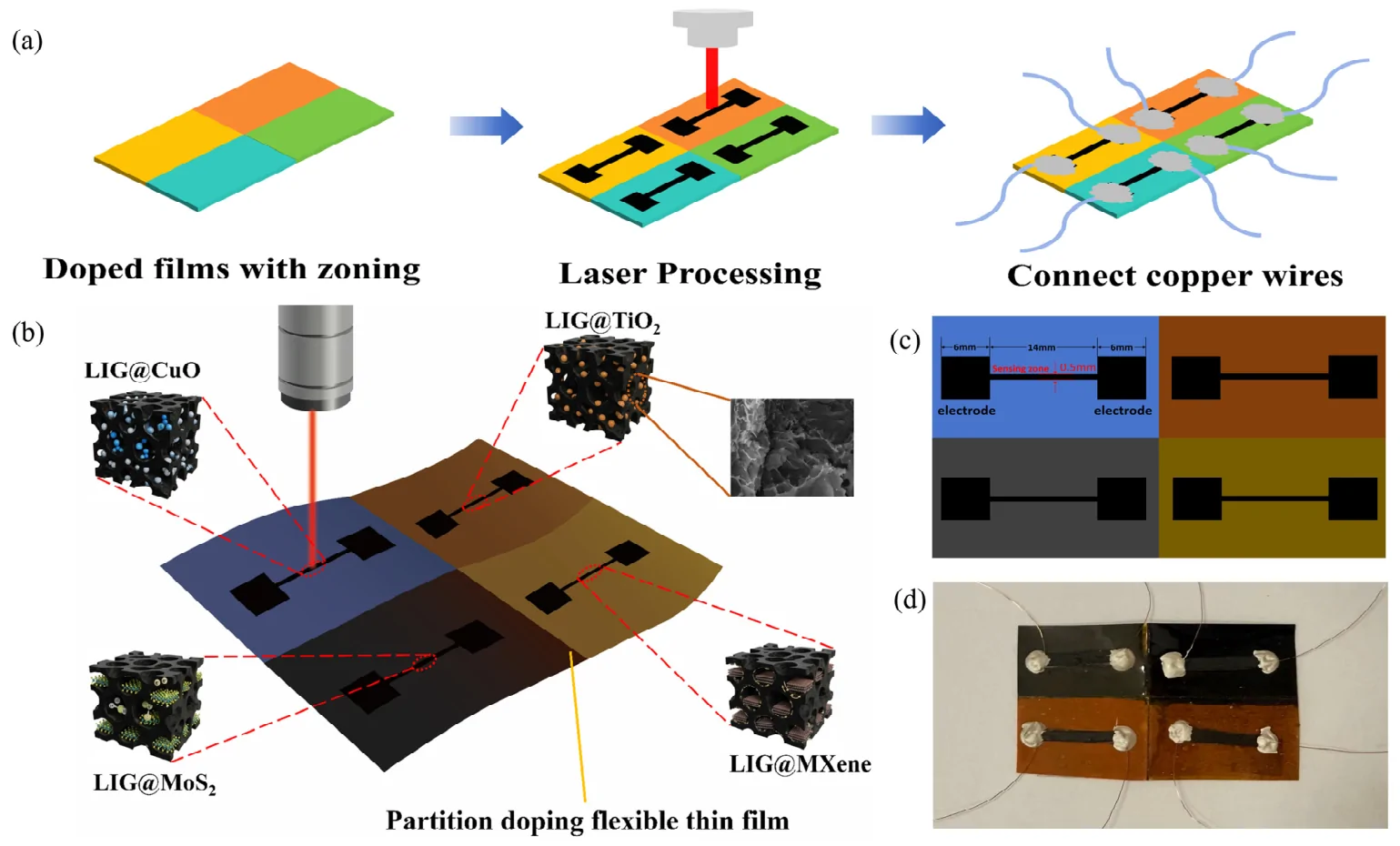
(**a**) Array sensor processing flow chart. (**b**) Laser direct writing to generate LIG-based composite schematic diagram. (**c**) The schematic diagram of array sensing design. (**d**) Photograph of the fabricated sensor array.

The fabrication process of the gas sensor array is illustrated in Figure 1a and described as follows:

(1) The prepared flexible partition-doped film (the preparation process is shown in Figure S1) was used as the substrate. The film consisted of four functional regions doped with different gas-sensitive materials, providing the basis for differentiated gas responses from the sensor array.
(2) According to the structure design of the array sensor, laser processing is carried out on the surface of each partition. Using the optimized laser power and scanning speed, the PI surface was locally carbonized to form LIG patterns in situ. In each functional region, a sensing unit composed of two-terminal electrodes and an intermediate sensitive channel is processed, in which the LIG structure serves as both a conductive network and an effective sensitive interface for gas adsorption and resistance change.
(3) After laser processing, copper wires were connected to the electrode regions of each sensing unit to establish electrical connections between the sensor array and the external measurement circuit. One end of each copper wire was placed on the corresponding LIG electrode region and fixed using conductive silver adhesive (Z-6011, Zhongshuo, Zhuhai, China). The adhesive was cured at room temperature for approximately 6 h to ensure stable electrical contact and reliable signal transmission. Finally, a flexible gas sensor array composed of four independent sensing units was obtained.

A flexible LIG-based gas sensor array was designed using a partition-doped film containing four independent sensing units functionalized with different gas-sensitive materials to generate differential responses toward multiple gas species. The LIG conductive pattern was formed on the surface of each detection unit through laser direct writing, as shown in Figure 1b. The wide areas at both ends served as electrodes, and the narrow area in the middle served as a gas-sensitive channel. As shown in Figure 1c, the electrode width of a single sensing unit was 6 mm, the length of the sensitive channel was 14 mm, and the channel width was 0.5 mm. This configuration provided stable electrical connections while maintaining sufficient exposure of the sensitive channel to the target gases. The differentiated responses generated by the four functionalized sensing units provide multidimensional features for subsequent gas identification. Figure 1d shows a photograph of the fabricated sensor array.

### 2.2. Gas-Sensing Measurements

Gas-sensing measurements were conducted using a dynamic gas distribution system (DGD-VI, Beijing Zhongju Gaoke Technology Co., Ltd., Beijing, China) equipped with mass flow controllers (MFCs), as shown in Figure S2. Synthetic air composed of 79% N_2_ and 21% O_2_ was used as the background gas, and the purities of the background gas and all target gases (Beijing Haipu Gas Co., Ltd., Beijing, China) were 99.999%. The total gas flow rate was maintained at 1000 sccm, and the effective internal volume of the test chamber was ~500 cm^3^. All measurements were performed at 25 °C and ~30% RH. The electrical signals of the sensing units were continuously recorded using a CGS-8R Gas Sensor Array Testing System under an applied bias voltage of 1 V, with the measured current in the range of approximately 0.2–10 mA. The dynamic response curves were smoothed using a Savitzky–Golay filter with a window length of 9 data points and a third-order polynomial to reduce high-frequency noise while preserving the overall response profile.

## 3. Results and Discussion

### 3.1. Laser Optimization

To establish appropriate femtosecond-laser processing conditions for the LIG-based composites, representative processing parameters reported for PI-based LIG systems were first compared, as summarized in Table S1 [20,21,22,23,24]. The reported conditions vary in laser wavelength, pulse duration, repetition rate, and power, indicating that LIG formation is governed by the combined laser-processing conditions rather than by laser power alone.

In order to optimize the laser processing parameters, the effects of laser power and scanning speed on LIG formation were systematically investigated. Laser power has a significant effect on the degree of carbonization, defect characteristics, and surface morphology of LIG [20,24]. All other processing parameters were kept constant: the repetition frequency was 200 kHz, the scanning speed was 9 mm/s, and the defocus distance was 0 mm. For the laser-power study, the laser power was varied from 300 mW to 600 mW. Raman spectroscopy was used to evaluate the graphitization and defect characteristics of the four LIG-based composites. The I_D_/I_G_ ratio provides information on the defect density and graphitic ordering of LIG, whereas the I_2D_/I_G_ ratio can be used as a qualitative indicator of the graphene-layer characteristics [25].

**Figure 2:**
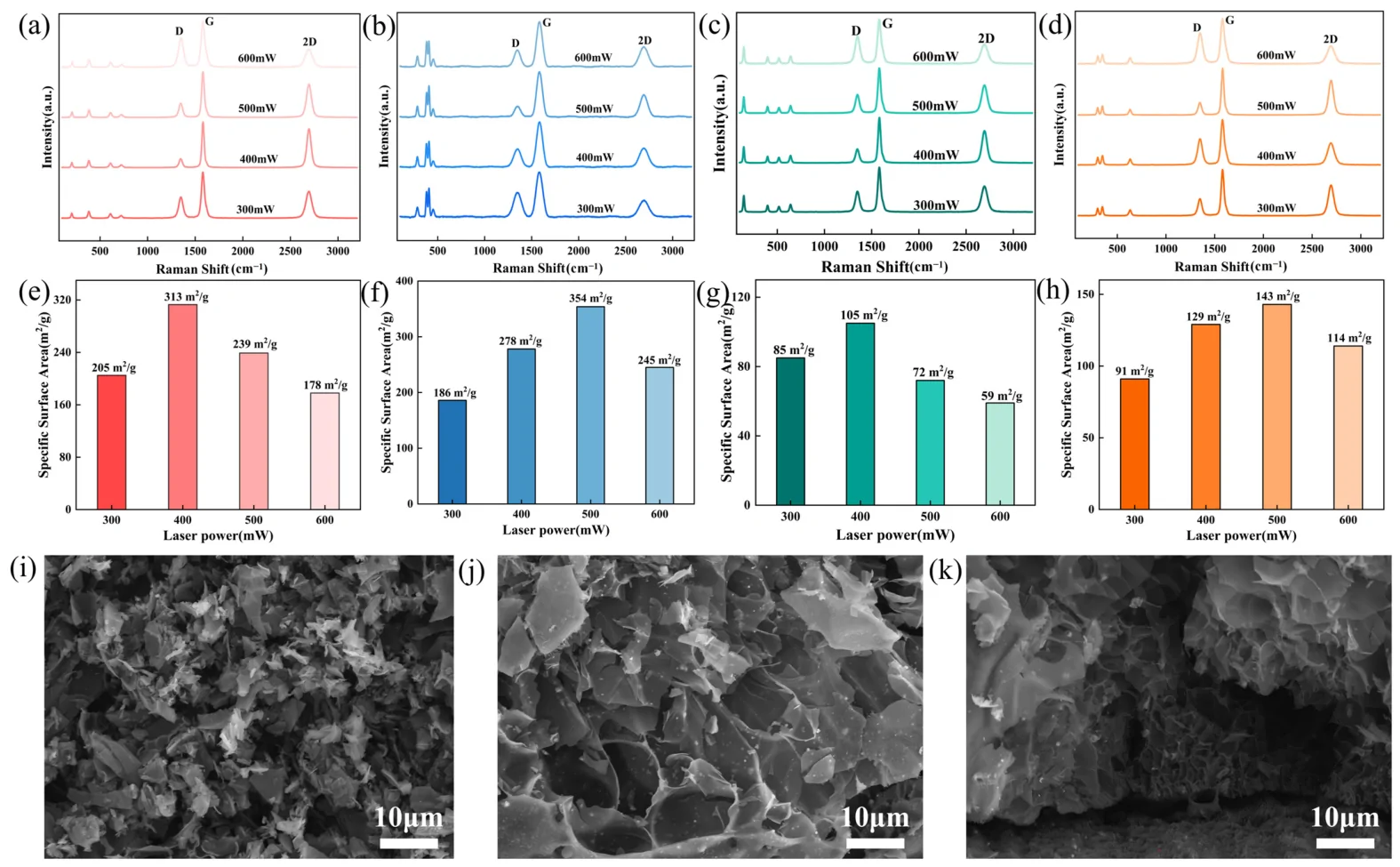
Laser-parameter optimization of LIG-based composites: power-dependent Raman spectra of (**a**) LIG@MXene, (**b**) LIG@MoS_2_, (**c**) LIG@TiO_2_, and (**d**) LIG@CuO; BET results of (**e**) LIG@MXene, (**f**) LIG@MoS_2_, (**g**) LIG@TiO_2_, and (**h**) LIG@CuO; SEM images of LIG@MXene prepared at scanning speeds of (**i**) 6, (**j**) 9, and (**k**) 12 mm s^−1^.

As shown in Figure 2a–d, the four composite systems exhibit different optimal laser powers. LIG@MXene and LIG@TiO_2_ show the lowest D-band intensity and relatively pronounced 2D bands at 400 mW, indicating improved graphitic ordering under this processing condition. In contrast, LIG@MoS_2_ and LIG@CuO exhibit their most favorable Raman characteristics at 500 mW. Accordingly, 400 mW was selected as the optimal laser power for LIG@MXene and LIG@TiO_2_, whereas 500 mW was selected for LIG@MoS_2_ and LIG@CuO. BET analysis was then performed to further evaluate the influence of laser power on the porous structure of the four LIG-based composites. The specific surface area of LIG is strongly dependent on the laser-processing conditions, reflecting changes in pore formation and structural development [26]. As shown in Figure 2e–h, the specific surface area exhibited a non-monotonic dependence on laser power. For LIG@MXene, the specific surface area increased from 205 m^2^ g^−1^ at 300 mW to 313 m^2^ g−1 at 400 mW, and then decreased to 239 and 178 m^2^ g^−1^ at 500 and 600 mW, respectively. LIG@MoS_2_ reached its maximum specific surface area of 354 m^2^ g^−1^ at 500 mW, whereas LIG@TiO_2_ exhibited a maximum value of 105 m^2^ g^−1^ at 400 mW. LIG@CuO reached its highest specific surface area of 143 m^2^ g^−1^ at 500 mW. These results were consistent with the Raman-based optimization and further supported the selection of 400 mW for LIG@MXene and LIG@TiO_2_, and 500 mW for LIG@MoS_2_ and LIG@CuO. Scanning speed was subsequently optimized because it directly affects the laser–material interaction time and energy deposition per unit length, thereby influencing the morphology and pore development of LIG [27]. LIG@MXene samples were fabricated at scanning speeds of 6, 9, and 12 mm s^−1^ while maintaining the repetition frequency at 200 kHz, the laser power at 400 mW, and the defocus distance at 0 mm. As shown in Figure 2i–k, pronounced differences in surface morphology were observed at different scanning speeds. At 6 mm s^−1^, the LIG exhibited a relatively irregular and locally damaged porous structure, indicating excessive local energy deposition at the lower scanning speed. At 9 mm s^−1^, a more continuous, open, and interconnected porous network was obtained. When the scanning speed was increased to 12 mm s^−1^, the porous structure became less developed, which can be attributed to the reduced energy deposition associated with the shorter laser–material interaction time. Therefore, 9 mm s^−1^ was selected as the scanning speed for subsequent sensor fabrication.

### 3.2. Structural Characterization

**Figure 3:**
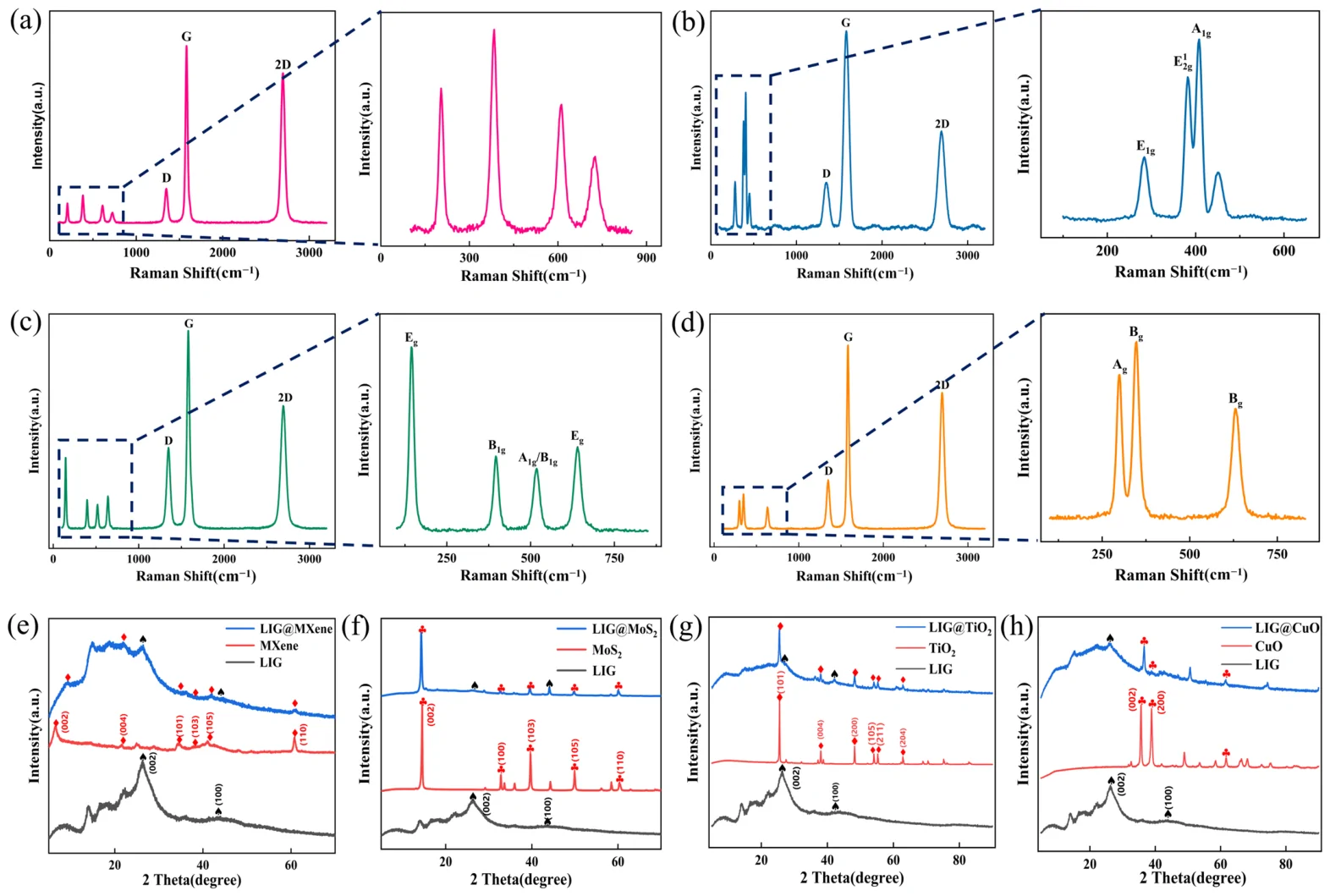
Structural characterization of LIG-based composites under optimized processing conditions: (**a**–**d**) Raman spectra of LIG@MXene, LIG@MoS_2_, LIG@TiO_2_, and LIG@CuO, respectively, with enlarged low-wavenumber regions indicated by the blue dashed boxes and connecting lines; (**e**–**h**) corresponding XRD patterns.

After optimization of the laser-processing parameters, the structural characteristics of the four LIG-based composites were further investigated by Raman spectroscopy and XRD. As shown in Figure 3a–d, all four composites exhibited the characteristic D, G, and 2D bands of LIG at approximately 1350, 1580, and 2700 cm^−1^, respectively, demonstrating the formation of graphene-like carbon structures after femtosecond-laser processing [25]. In addition to the carbon-related bands, characteristic Raman features of the corresponding functional phases were observed in the enlarged low-wavenumber regions. LIG@MXene exhibited several characteristic vibrational features in the low-wavenumber region associated with Ti_3_C_2_T_x_ MXene [28]. LIG@MoS_2_ exhibited the characteristic E_1g_, E_2g_^1^, and A_1g_ vibration modes of MoS_2_ [29]. LIG@TiO_2_ showed the characteristic E_g_, B_1g_, A_1g_/B_1g_, and E_g_ modes of anatase TiO_2_ [30], whereas LIG@CuO exhibited the characteristic A_g_ and B_g_ vibration modes of CuO [31]. These results demonstrate that the four composites retained both the characteristic carbon structure of LIG and the Raman features of the corresponding functional phases. The XRD patterns in Figure 3e–h further confirmed the coexistence of the LIG framework and the corresponding functional phases. As shown in Figure 3e, LIG@MXene retained the characteristic low-angle (002) diffraction feature of MXene, together with the broad LIG-related diffraction contribution near 26°, indicating the coexistence of MXene and graphene-like carbon structures [32,33]. In Figure 3f, LIG@MoS_2_ exhibited the characteristic diffraction peaks of layered MoS_2_, including the prominent (002) reflection, while the diffraction features of LIG remained observable [34]. As shown in Figure 3g, LIG@TiO_2_ displayed the characteristic reflections of anatase TiO_2_, including the (101) and (004) planes, together with the broad diffraction contribution of LIG [35]. Similarly, the characteristic diffraction peaks of CuO were retained in the LIG@CuO composite, as shown in Figure 3h, while the LIG-related diffraction features were also present [36]. Overall, the XRD results provide complementary evidence for the successful formation of the four LIG-based composites and the preservation of the corresponding functional crystalline phases.

### 3.3. Gas-Sensing Performance

**Figure 4:**
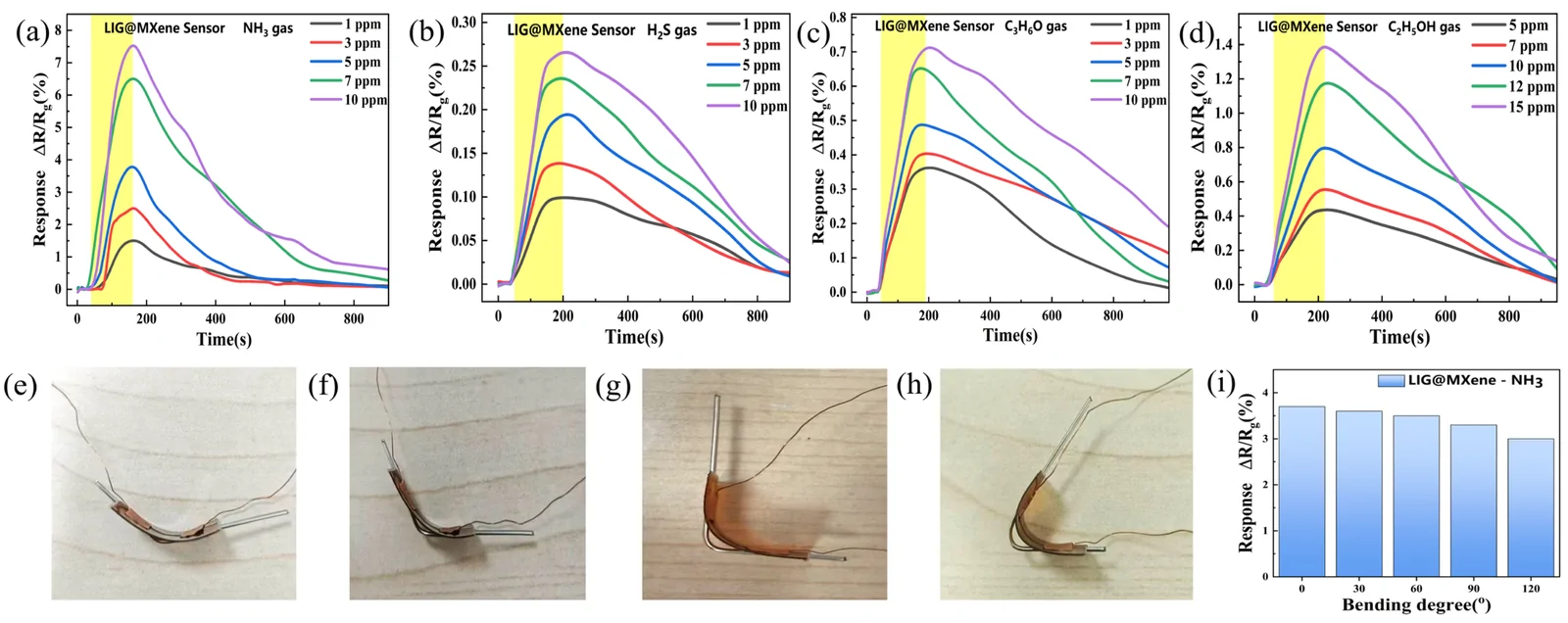
Gas-sensing and flexibility characteristics of the LIG@MXene sensing unit: (**a**–**d**) concentration-dependent responses toward NH_3_, H_2_S, C_3_H_6_O, and C_2_H_5_OH, respectively, with the yellow shaded regions indicating the gas exposure periods; (**e**) 30°, (**f**) 60°, (**g**) 90°, and (**h**) 120° bending states of the sensor; (**i**) NH_3_ response of the LIG@MXene sensing unit under different bending angles.

Figure 4a–d show the concentration-dependent responses of the LIG@MXene sensing unit toward NH_3_, H_2_S, C_3_H_6_O, and C_2_H_5_OH, respectively. NH_3_, H_2_S, and C_3_H_6_O were tested at concentrations of 1, 3, 5, 7, and 10 ppm, whereas C_2_H_5_OH was tested at 5, 7, 10, 12, and 15 ppm. For all four gases, the response amplitude increased with increasing gas concentration, which can be attributed to the increased adsorption of gas molecules on accessible sensing sites and the resulting enhancement of gas-induced charge transfer. The optimized LIG@MXene composite possessed a porous conductive network and relatively high accessible surface area, providing abundant adsorption sites and efficient diffusion pathways for gas molecules. Meanwhile, the coexistence of the LIG conductive framework and the MXene functional phase provides an interfacial structure favorable for charge-transfer modulation during gas adsorption. Among the four gases, LIG@MXene exhibited a substantially stronger response toward NH_3_, and a clear response was still observed at 1 ppm. This preferential response can be associated with the interaction between NH_3_ molecules and the surface terminations of Ti_3_C_2_T_x_ MXene, which facilitate molecular adsorption and charge transfer at room temperature [37]. The porous LIG network provides a continuous conductive pathway, while interfacial charge redistribution between MXene and LIG further modulates the electrical response. The corresponding concentration-dependent responses of LIG@MoS_2_, LIG@TiO_2_, and LIG@CuO toward the four target gases are provided in Figures S3–S5, respectively.

The mechanical flexibility of the sensing unit was further evaluated under different bending conditions, as shown in Figure 4e–i. The device was bent to 30°, 60°, 90°, and 120°, while its NH_3_ sensing performance was measured under the same testing conditions. Under 5 ppm NH_3_, the response gradually decreased from approximately 3.68% in the flat state to 3.10% at a bending angle of 120°, corresponding to a response retention of approximately 84.2%. Although a moderate decrease in response was observed with increasing bending angle, the sensor maintained a clear and distinguishable gas response throughout the investigated deformation range. This behavior indicates that the flexible PI substrate and interconnected porous LIG conductive network can maintain effective electrical pathways under mechanical deformation, thereby preserving the basic gas-sensing function of the device. These results demonstrate the mechanical adaptability of the LIG@MXene sensing unit for flexible sensing applications.

**Figure 5:**
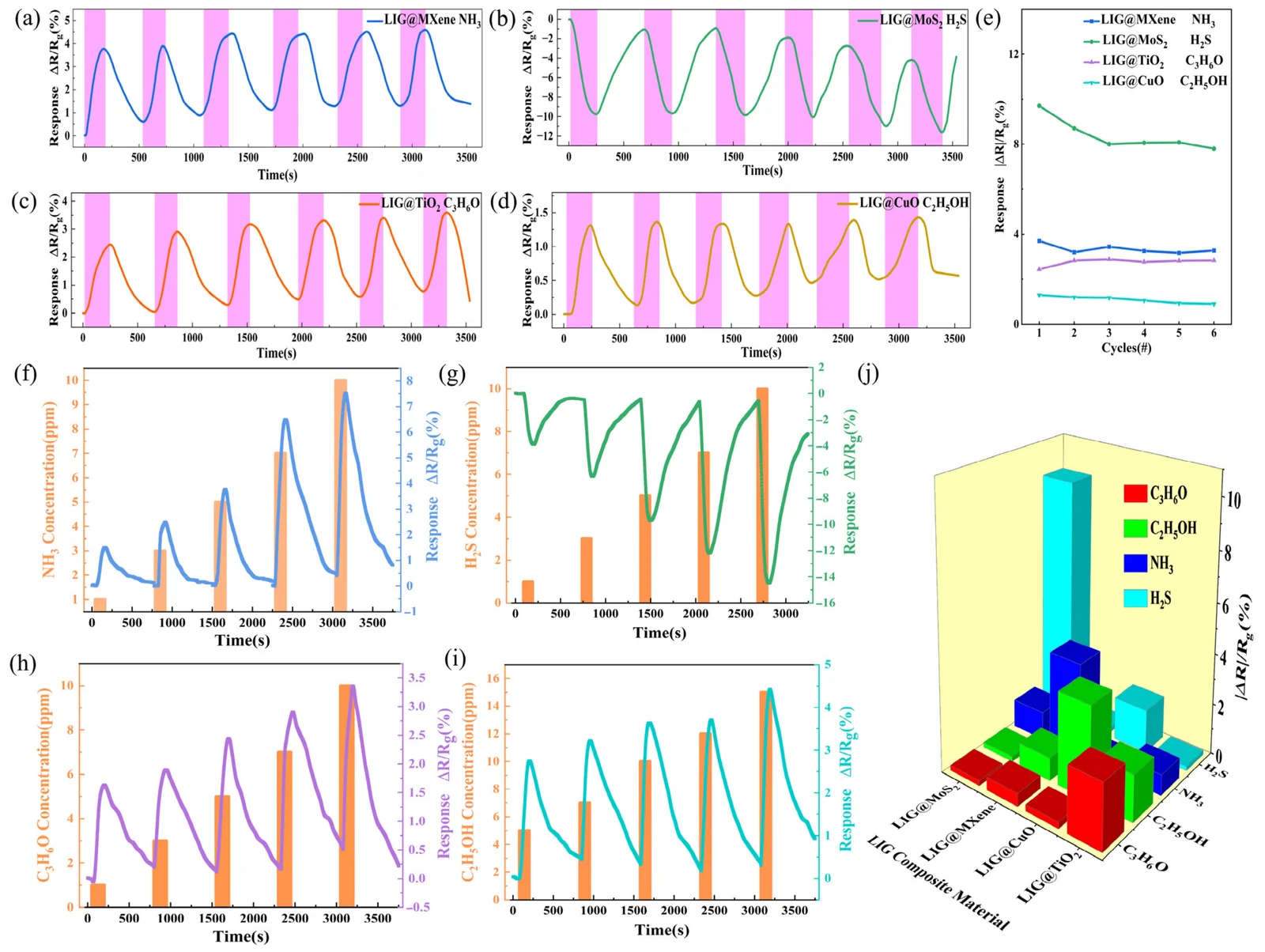
Gas-sensing performance of the four functionalized LIG-based sensing units: (**a**–**d**) cycle responses to NH_3_, H_2_S, C_3_H_6_O, and C_2_H_5_OH, respectively; (**e**) response stability over cycles; (**f**–**i**) concentration-dependent responses toward the corresponding target gases; (**j**) differentiated responses of the four sensing units toward the four target gases.

Figure 5a–d show the cyclic response–recovery behavior of the four functionalized LIG-based sensing units toward their corresponding target gases. As shown in Figure 5a, LIG@MXene exhibited reproducible positive responses toward NH_3_, with similar response profiles during repeated gas exposure and purge cycles. In contrast, LIG@MoS_2_ showed a characteristic negative response toward H_2_S, as shown in Figure 5b, and the response magnitude gradually decreased during the initial cycles before approaching a relatively stable level. LIG@TiO_2_ exhibited repeatable positive responses toward C_3_H_6_O with relatively stable response amplitudes over consecutive cycles (Figure 5c). Similarly, LIG@CuO showed reproducible positive responses toward C_2_H_5_OH, although a certain degree of recovery tailing was observed between successive cycles (Figure 5d). Overall, the four sensing units maintained distinguishable and repeatable response characteristics toward their corresponding target gases, while exhibiting material-dependent differences in response direction and recovery behavior. Figure 5e summarizes the response amplitudes of the four sensing units during repeated gas-exposure cycles. LIG@MoS_2_ exhibited the largest response magnitude toward H_2_S, although its response gradually decreased during the initial cycles and subsequently approached a relatively stable level. In comparison, LIG@MXene and LIG@TiO_2_ showed relatively small cycle-to-cycle variations toward NH_3_ and C_3_H_6_O, respectively. LIG@CuO exhibited a lower response amplitude toward C_2_H_5_OH together with a slight decrease during repeated exposure. These results indicate that all four sensing units retained repeatable sensing responses, although their response magnitude and short-term cycling stability were dependent on the functional material and target gas.

Figure 5f–i further show the dynamic responses of the four sensing units under progressively increasing concentrations of their corresponding target gases. For LIG@MXene, the positive response to NH_3_ increased progressively as the concentration increased from 1 to 10 ppm (Figure 5f). In contrast, LIG@MoS_2_ exhibited increasingly pronounced negative responses toward H2S with increasing concentration (Figure 5g). LIG@TiO_2_ also showed an increasing positive response toward C_3_H_6_O over the investigated concentration range (Figure 5h), while LIG@CuO exhibited a similar concentration-dependent increase in response toward C_2_H_5_OH (Figure 5i). The systematic variation in response magnitude with gas concentration demonstrates that the four functionalized sensing units preserve their characteristic response directions while providing concentration-dependent sensing signals.

Figure 5j further compares the differentiated responses of the four functionalized sensing units toward the four target gases. Each gas produced a distinct combination of response amplitudes across the four sensing channels rather than an exclusive response from a single sensing unit. LIG@MXene exhibited a preferential response toward NH_3_. Ti_3_C_2_T_x_ MXene possesses abundant surface terminations, including -O, -OH, and-F groups, which provide active adsorption sites for polar NH_3_ molecules. The adsorption and electron-donating behavior of NH_3_ can modulate the carrier concentration and interfacial charge distribution in the LIG@MXene composite. The observed increase in resistance is consistent with a p-type-like response of the composite conductive network, while the porous LIG framework provides continuous conductive pathways and facilitates gas access to the MXene-active interfaces [37]. LIG@MoS_2_ showed the most pronounced negative response toward H2S. Owing to the n-type semiconducting characteristics of MoS_2_, H_2_S adsorption can increase the electron concentration and enhance electron transport, resulting in a decrease in resistance. Interfacial charge redistribution between MoS_2_ and LIG can further modulate the electrical response [38]. For LIG@TiO_2_, the positive response toward C_3_H_6_O can be attributed to the combined effects of the TiO_2_ functional phase, the LIG conductive network, and interfacial carrier modulation. Carbon/TiO_2_ composite systems can exhibit p-type-like resistance changes at room temperature, indicating that the carbon network and heterointerface can strongly influence the overall transport behavior [39]. Acetone adsorption therefore modulates carrier transport in the LIG@TiO2 composite, resulting in the observed increase in resistance. LIG@CuO also exhibited a positive response toward C_2_H_5_OH. As CuO is a p-type semiconductor, ethanol-induced charge transfer can reduce the effective hole concentration and increase the resistance of the sensing channel, while the LIG conductive network and LIG/CuO interface further contribute to the overall electrical modulation [40]. Therefore, the different surface chemistries, majority-carrier characteristics, and gas-induced charge-transfer processes of MXene, MoS_2_, TiO_2_, and CuO, together with the porous conductive LIG framework and interfacial charge redistribution, give rise to distinct response amplitudes and polarities across the four channels. These differentiated multi-channel response patterns provide the multidimensional sensing features required for subsequent BPNN-based gas identification.

### 3.4. Gas Identification

To identify single gases and mixed-gas combinations, a BPNN was employed based on the differential multi-channel responses of the flexible 2 × 2 gas sensor array. Machine-learning-assisted analysis of multidimensional sensing signals provides an effective approach for gas discrimination in sensor-array systems [19,41]. The BPNN performed discrete gas-condition identification using predefined numerical labels. The response amplitudes of the four sensing units were used as four input features and mapped to predefined numerical labels as target outputs. The trained network generated a continuous numerical output corresponding to the encoded target label. A BPNN is a multilayer feedforward neural network trained using the error backpropagation algorithm. The network consisted of an input layer with four neurons, one hidden layer with eight neurons, and one output neuron.

In the single-gas identification experiment, 20 labels were defined based on four gas species and five concentration levels, as shown in Table S2. At room temperature, when a single gas at a given concentration was introduced into the gas chamber, the four gas-sensitive regions generated four corresponding maximum response values. These four response values were recorded as the input features of each labeled sample. Four repeated measurements were performed for each label, resulting in a total of 20 × 4 = 80 samples.

During model training, the data set was randomly divided into three subsets, including 58 samples for training, 11 samples for validation, and 11 samples for testing, corresponding to an approximate ratio of 70%:15%:15%. The training set was used to optimize the network parameters, the validation set was used to monitor the training process and model performance, and the test set was used to evaluate the trained model.

**Figure 6:**
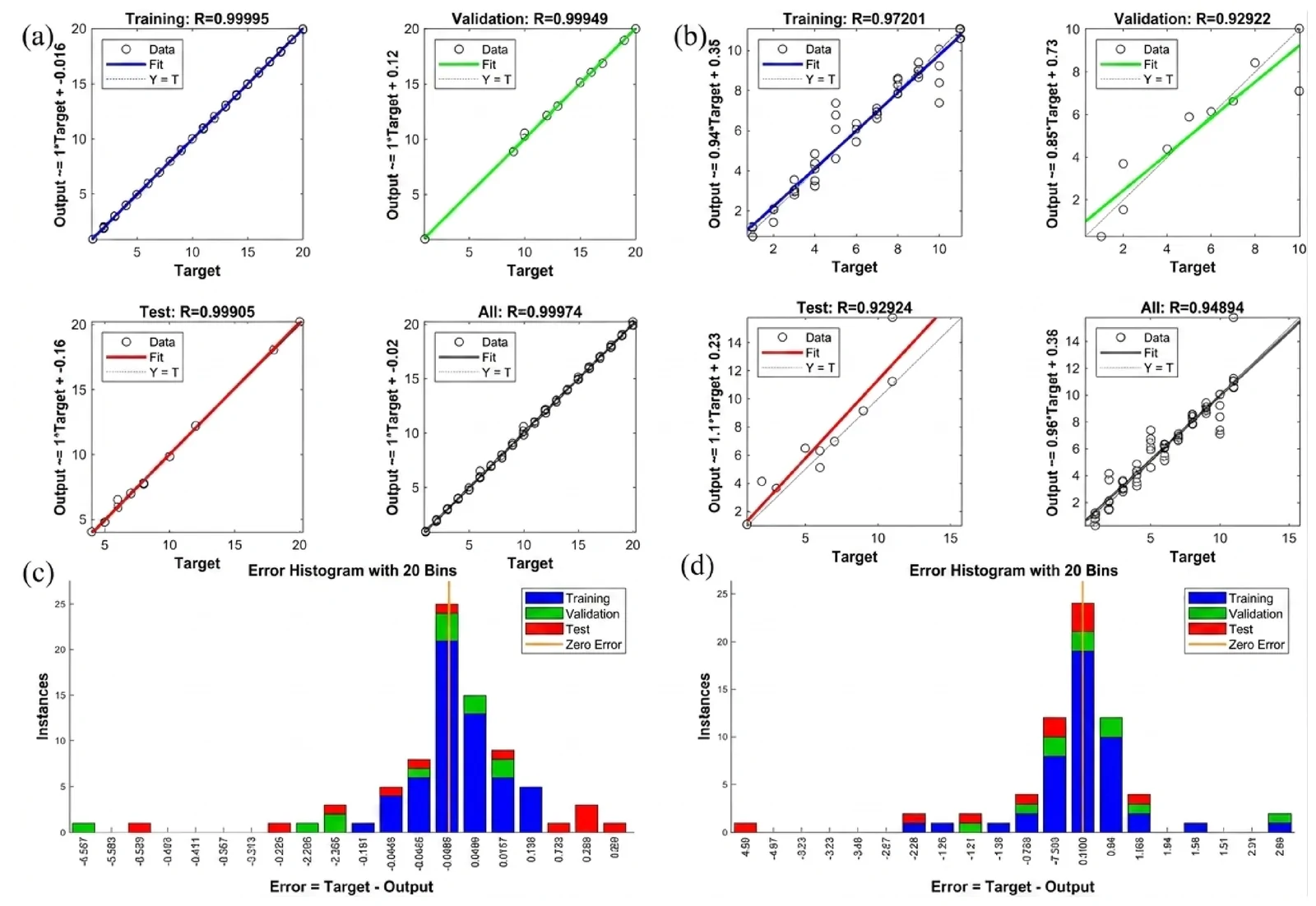
BPNN-based gas identification results: (**a**) target–output correlation for single-gas conditions; (**b**) target–output correlation for mixed-gas combinations; (**c**) error distribution for single-gas identification; and (**d**) error distribution for mixed-gas identification.

The target–output correlation results of the single-gas identification model for the training, validation, test, and overall datasets are shown in Figure 6a. The corresponding correlation coefficients were 0.99995, 0.99949, 0.99905, and 0.99974, respectively, indicating a strong linear correlation between the network outputs and the numerically encoded target labels. The straight fitted lines shown in Figure 6a were obtained by post hoc linear regression between the BPNN outputs and the encoded target values to visualize their correspondence. The fitted lines are close to the ideal Y = T line, supporting good correspondence between the network outputs and the corresponding target labels.

Figure 6c shows the error distribution histogram of the single-gas identification model. Here, the error is defined as the difference between the numerically encoded target value and the corresponding network output. The error is distributed between −0.6 and +0.3, and the sample error is mainly concentrated near zero, with the peak interval in the range of approximately 0.01–0.04. This result indicates that most network outputs are close to their corresponding target labels. The error distributions of the training, validation, and test sets show similar trends, suggesting relatively consistent model performance across the different data subsets.

In practical application scenarios, multiple gases may coexist simultaneously. Therefore, mixed-gas experiments were further conducted to evaluate the ability of the sensor array to distinguish different gas combinations. Eleven labels were defined for different combinations containing two, three, or four gases, as shown in Table S3. The concentrations of NH_3_, H_2_S, C_3_H_6_O, and C_2_H_5_OH were fixed at 5 ppm, 5 ppm, 5 ppm, and 10 ppm, respectively. Under each mixed-gas condition, the four gas-sensitive regions generated four corresponding response values, which were used as the input features for mixed-gas identification. Six repeated measurements were performed for each of the 11 mixed-gas labels, resulting in a total of 11 × 6 = 66 samples. The mixed-gas data set was randomly divided into 46 training samples, 10 validation samples, and 10 test samples.

The target–output correlation results of the mixed-gas identification model are shown in Figure 6b. The correlation coefficients of the training, validation, test, and overall datasets were R = 0.97201, R = 0.92922, R = 0.92924, and R = 0.94894, respectively. These correlation coefficients characterize the linear relationship between the numerical network outputs and the encoded target values. Although the correlation coefficients are lower than those obtained for the single-gas dataset, the network outputs still show an overall correspondence with the encoded labels for the investigated mixed-gas combinations.

Figure 6d shows the error distribution histogram of the mixed-gas identification model. The overall error distribution ranges from −4.59 to +2.699, and the error is mainly concentrated near zero, with the highest-frequency interval located around 0.001. Compared with the single-gas identification results, the broader error distribution reflects the increased complexity of distinguishing mixed-gas combinations. The similar error-distribution trends observed for the training, validation, and test subsets indicate that comparable output-error characteristics are maintained across the different data subsets.

## 4. Conclusions

A flexible 2 × 2 multi-gas sensing array based on region-functionalized LIG composites was developed for room-temperature detection and identification of NH_3_, H_2_S, acetone, and ethanol. Selective incorporation of MXene, MoS_2_, TiO_2_, and CuO produced four sensing channels with distinct response amplitudes and polarities, while the porous LIG network provided conductive pathways and accessible gas-sensitive interfaces. The sensor array exhibited repeatable cyclic responses, clear concentration-dependent behavior, and retained approximately 84.2% of its NH_3_ response at a bending angle of 120°. The differentiated four-channel responses were further used for BPNN-based gas-condition identification, showing good correspondence between the network outputs and the encoded target labels for both single-gas and mixed-gas datasets. These results demonstrate the feasibility of integrating region-specific functionalization, femtosecond-laser direct writing, flexible multi-channel sensing, and intelligent gas identification within a single LIG-based platform. Future work will focus on humidity-dependent sensing behavior and environmental robustness under practical operating conditions.

## Data Availability

The data presented in this study are available from the corresponding author upon reasonable request. The data are not publicly available due to privacy restrictions.

## Supplementary Materials

The following supporting information can be downloaded at: https://www.mdpi.com/article/10.3390/nano16201321/s1, Figure S1: Preparation of flexible partition doped films; Figure S2: Gas sensor test platform (a) test system; (b) Cylinder; (c) gas distribution system; (d) Test chamber; Figure S3: The gas response curves of different concentrations of different kinds of gas (a) NH_3_ were tested by LIG@MoS_2_ sensor. (b) H_2_S; (c) C_3_H_6_O; (d) C_2_H_5_OH; Figure S4: The LIG@TiO_2_ sensor was used to test the gas response curves of different concentrations of different gases (a) NH_3_; (b) H_2_S; (c) C_3_H_6_O; (d) C_2_H_5_OH; Figure S5: The LIG@CuO sensor tests the gas response curves of different concentrations of different kinds of gases (a) NH_3_; (b) H_2_S; (c) C_3_H_6_O; (d) C_2_H_5_OH; Table S1: Comparison of representative femtosecond-laser processing conditions for PI-based LIG; Table S2: Single gas classification test label; Table S3: Multi-gas test label.
