# Supplementary material for: Region-Functionalized Laser-Induced Graphene Sensor Array for Flexible and Intelligent Multi-Gas Detection
Source: Nanomaterials (Basel). 2026 Oct 20;16(20):1321. doi: 10.3390/nano16201321 (PMC13708456; doi:10.3390/nano16201321)
Supplement: Supplementary file 1 [file nanomaterials-16-01321-s001.zip › nanomaterials-4537855-supplementary.pdf]

Region-Functionalized Laser-Induced Graphene Sensor Array for Flexible and Intelligent Multi-Gas Detection

Wengan Wang, Chaoqi Zhang, Guiyong Chen, PeiHao Xu, Fan Wu, Misheng Liang*, Rui You*

1 AVIC Chengdu Aircraft Industrial (Group) Co., Ltd., Chengdu 610092, China

2 School of Instrument Science and Opto-Electronics Engineering, Beijing Information Science and Technology University, Beijing 100192,China

3 Laboratory of Intelligent Microsystems, Beijing Information Science and Technology University, Beijing 100192, China

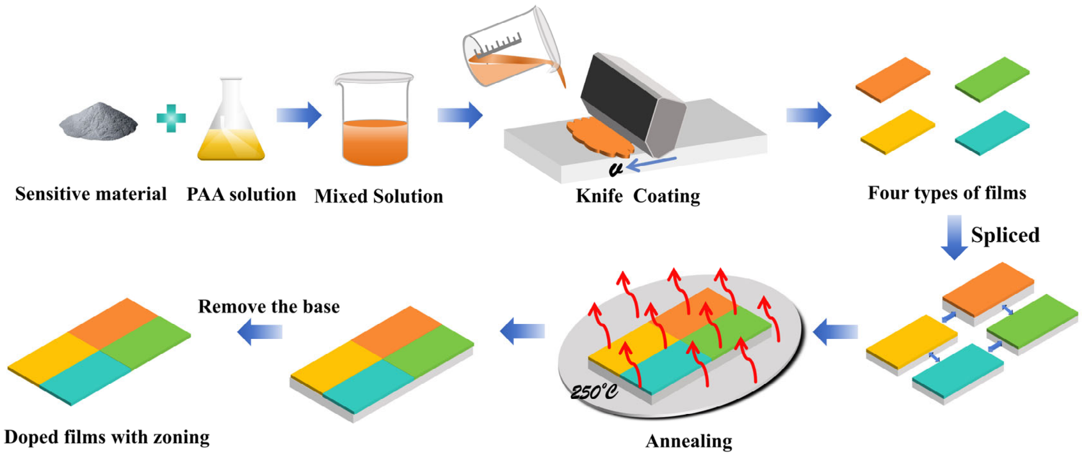

Figure S1 Preparation of flexible partition doped films

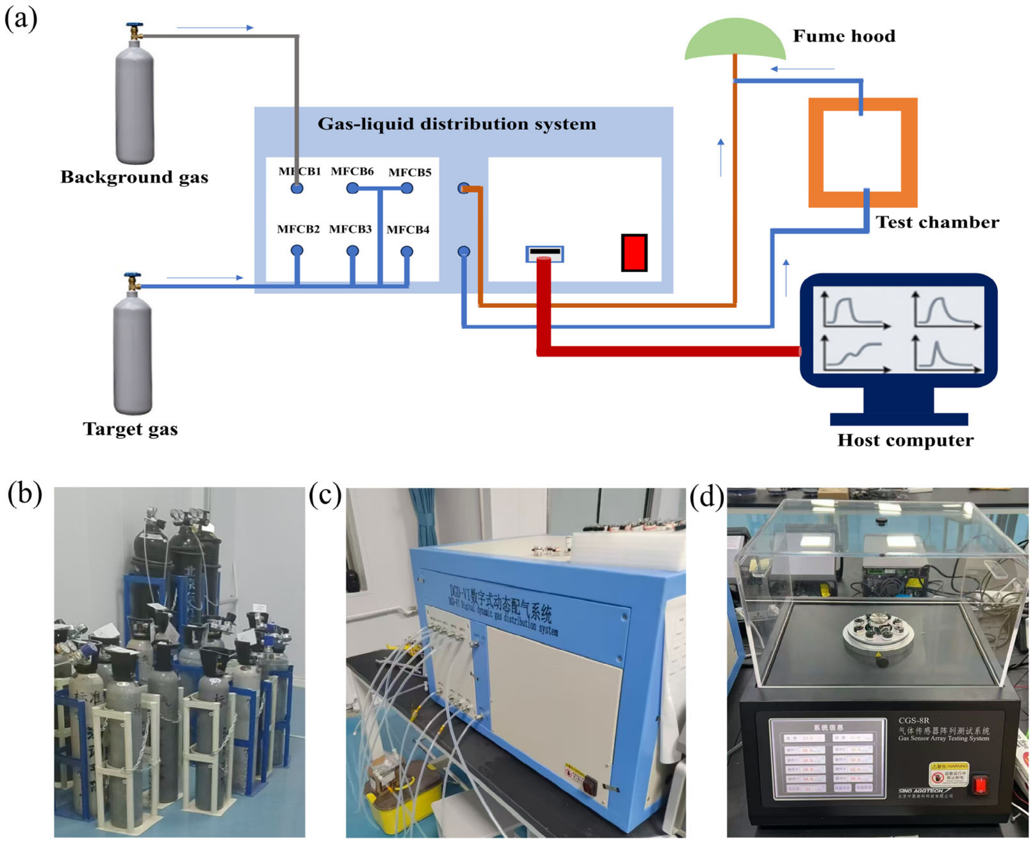

Figure S2 Gas sensor test platform (a) test system ; (b) Cylinder ; (c) gas distribution system ; (d) Test chamber

Table S1. Comparison of representative femtosecond-laser processing conditions for PI-based LIG.

Sample	Wavelength (nm)	Pulse duration (fs)	Rep. rate (kHz)	Power (W)	Ref.
PI-based LIG	1030	550	200	0.242–0.281	[20]
CPI-based LIG	515	170	10	NR	[21]
PI–MXene-based LIG	1030	NR	500	2.0	[22]
PI-based LIG	1030	NR	700	1.3	[23]
PI-based LIG	1030	400	500	3.0	[24]
This work	1030	137	200	0.4–0.5	—

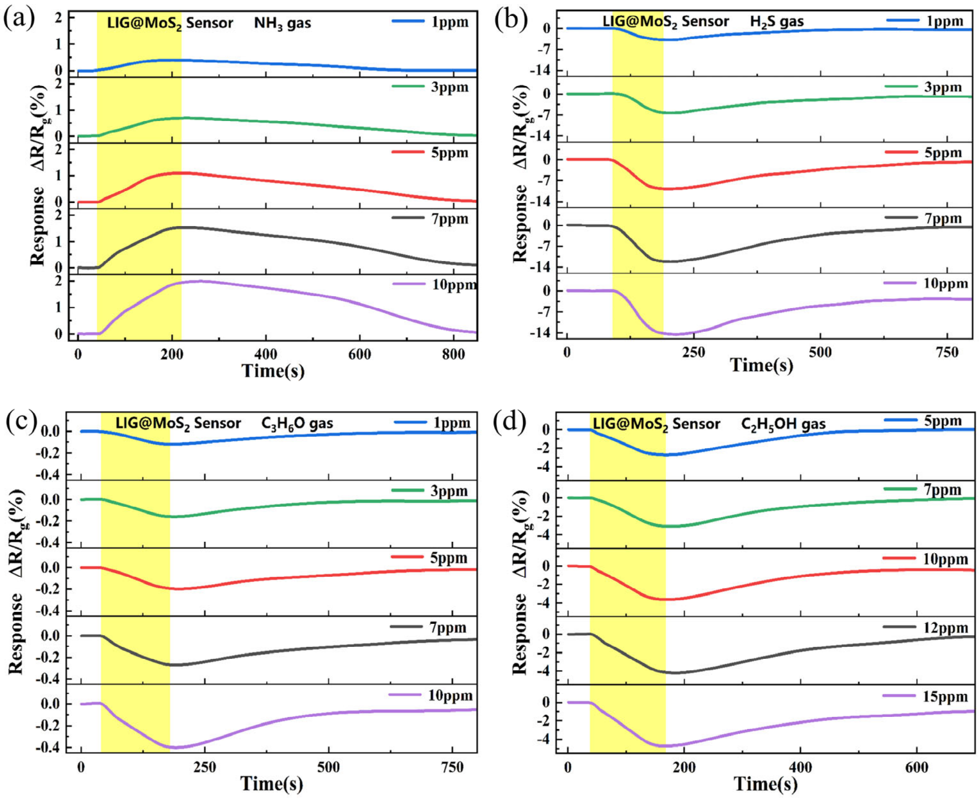

Figure S3 The gas response curves of different concentrations of different kinds of gas (a) NH_3 were tested by LIG@MoS_2 sensor. (b) H_2S ; (c) C_3H_6O ; (d) C_2H_5OH

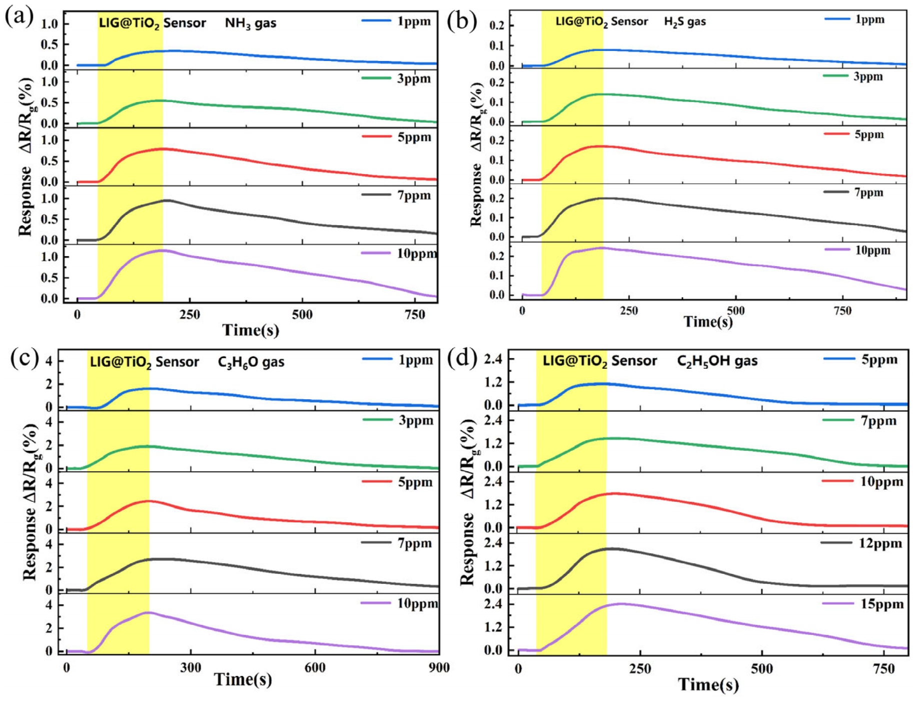

Figure S4 The LIG@TiO_2 sensor was used to test the gas response curves of different

concentrations of different gases (a) NH_3 ; (b) H_2S ; (c) C_3H_6O ; (d) C_2H_5OH

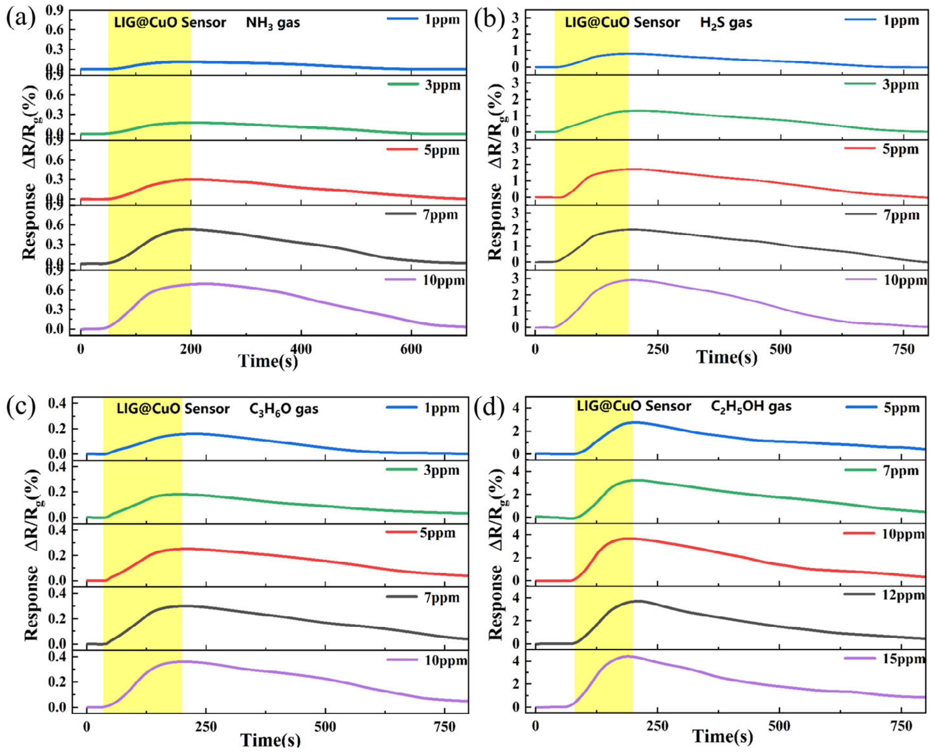

Figure S5 The LIG@CuO sensor tests the gas response curves of different concentrations of different kinds of gases (a) NH_3 ; (b) H_2S ; (c) C_3H_6O ; (d) C_2H_5OH

Table S2 Single gas classification test label

Label	Gas	Concentration
1	NH_3	1ppm
2	NH_3	3 ppm
3	NH_3	5 ppm
4	NH_3	7 ppm
5	NH_3	10 ppm
6	H_2S	1 ppm
7	H_2S	3 ppm
8	H_2S	5 ppm
9	H_2S	7 ppm
10	H_2S	10 ppm
11	C_3H_6O	1 ppm
12	C_3H_6O	3 ppm
13	C_3H_6O	5 ppm
14	C_3H_6O	7 ppm
15	C_3H_6O	10 ppm
16	C_2H_5OH	5 ppm
17	C_2H_5OH	7 ppm
18	C_2H_5OH	10 ppm
19	C_2H_5OH	12 ppm
20	C_2H_5OH	15 ppm

Table S3 Multi-gas test label

Number of gases	Label	Gas	Concentration (unit : ppm)	Proportion
	1	NH_3+H_2S	5+5	1:1
	2	NH_3+C_3H_6O	5+5	1:1
	3	NH_3+C_2H_5OH	5+10	1:2
2	4	H_2S+C_3H_6O	5+5	1:1
	5	H_2S+C_2H_5OH	5+10	1:2
	6	C_3H_6O+C_2H_5OH	5+10	1:2
	7	NH_3+H_2S+C_3H_6O	5+5+5	1:1:1
	8	NH_3+H_2S+C_2H_5OH	5+5+10	1:1:2
3	9	NH_3+C_3H_6O+C_2H_5OH	5+5+10	1:1:2
	10	H_2S+C_3H_6O+C_2H_5OH	5+5+10	1:1:2
4	11	NH_3+H_2S+C_3H_6O+C_2H_5OH	5+5+5+10	1:1:1:2
